# An observational study of surface versus endovascular cooling techniques in cardiac arrest patients: a propensity-matched analysis

**DOI:** 10.1186/s13054-015-0819-7

**Published:** 2015-03-16

**Authors:** Sang Hoon Oh, Joo Suk Oh, Young-Min Kim, Kyu Nam Park, Seung Pill Choi, Gi Woon Kim, Kyung Woon Jeung, Tae Chang Jang, Yoo Seok Park, Yeon Young Kyong, On behalf of the Korean Hypothermia Network Investigators

**Affiliations:** Department of Emergency Medicine, College of Medicine, The Catholic University of Korea, 222, Banpodaero, Seocho-gu, Seoul, Korea 137-701; Department of Emergency Medicine, College of Medicine, Ajou University, 164, World cup-ro, Yeongtong-gu, Suwon-si, Gyeonggi-do, Korea 443-380; Department of Emergency Medicine, College of Medicine, Chonnam National University, 42, Jebong-ro, Dong-gu, Gwangju, South Korea 501-757; Department of Emergency Medicine, College of Medicine, Catholic University of Daegu, 33, Duryugongwonro 17-gil, Nam-gu, Daegu, Korea 705-718; Department of Emergency Medicine, College of Medicine, Yonsei University, 50-1, Yonsei-ro, Seodaemun-gu, Seoul, Korea 120-752

## Abstract

**Introduction:**

Various methods and devices have been described for cooling after cardiac arrest, but the ideal cooling method remains unclear. The aim of this study was to compare the neurological outcomes, efficacies and adverse events of surface and endovascular cooling techniques in cardiac arrest patients.

**Methods:**

We performed a multicenter, retrospective, registry-based study of adult cardiac arrest patients treated with therapeutic hypothermia presenting to 24 hospitals across South Korea from 2007 to 2012. We included patients who received therapeutic hypothermia using overall surface or endovascular cooling devices and compared the neurological outcomes, efficacies and adverse events of both cooling techniques. To adjust for differences in the baseline characteristics of each cooling method, we performed one-to-one matching by the propensity score.

**Results:**

In total, 803 patients were included in the analysis. Of these patients, 559 underwent surface cooling, and the remaining 244 patients underwent endovascular cooling. In the unmatched cohort, a greater number of adverse events occurred in the surface cooling group. Surface cooling was significantly associated with a poor neurological outcome (cerebral performance category 3–5) at hospital discharge (p = 0.01). After propensity score matching, surface cooling was not associated with poor neurological outcome and hospital mortality [odds ratio (OR): 1.26, 95% confidence interval (CI): 0.81-1.96, p = 0.31 and OR: 0.85, 95% CI: 0.55-1.30, p = 0.44, respectively]. Although surface cooling was associated with an increased incidence of adverse events (such as overcooling, rebound hyperthermia, rewarming related hypoglycemia and hypotension) compared with endovascular cooling, these complications were not associated with surface cooling using hydrogel pads.

**Conclusions:**

In the overall matched cohort, no significant difference in neurological outcomes and hospital morality was observed between the surface and endovascular cooling methods.

**Electronic supplementary material:**

The online version of this article (doi:10.1186/s13054-015-0819-7) contains supplementary material, which is available to authorized users.

## Introduction

The International Liaison Committee on Resuscitation recommends the cooling of unconscious patients who present after having been resuscitated from out-of-hospital cardiac arrest (OHCA) at a temperature of 32 to 34°C for 12 to 24 hours [1,2]. Various methods, such as surface and core cooling, and various devices are described for administration of this therapy. Surface cooling methods include ice-pack application and water-circulating blankets/wraps/caps/helmets/gel pads [3-6]. Cold intravenous infusion and cooling with endovascular catheters or extracorporeal membrane oxygenation are examples of core cooling methods [7-11].

Among these methods, ice-pack application or cold fluid infusion are performed by staff without the aid of temperature feedback regulation and are currently used as adjuvant cooling. Surface cooling techniques that utilize automatic temperature feedback devices are generally considered less expensive and are the most widely used method. An additional widespread cooling method is feedback-controlled endovascular cooling [10]. A heat-exchange catheter placed in the vena cava ensures tight control of the core temperature by a temperature-guided control unit. The control unit automatically steers the cooling process as well as the maintenance and rewarming phase, and this unit has demonstrated improved temperature control. However, consensus has not been achieved regarding the ideal method for therapeutic hypothermia (TH). Several studies have compared cooling methods in TH after the return of spontaneous circulation [12-14]. However, these studies were limited due to few, uncontrolled data or a focus on comparisons between specific devices, thus not demonstrating a difference between surface and endovascular cooling techniques.

The aim of this study was to compare overall surface cooling methods with endovascular cooling methods with regard to the neurological outcome, cooling efficacy and adverse events of TH after cardiac arrest.

## Materials and methods

### Study design

This was a multicenter, retrospective, observational, registry-based study using the Korean Hypothermia Network registry data. The Korean Hypothermia Network, a multicenter clinical research consortium for TH in South Korea, was organized in 2011. Korean Hypothermia Network investigators collected data on adult (≥18 years) OHCA patients who received TH in 24 teaching hospitals in South Korea from 2007 to 2012. Patients who experienced traumatic cardiac arrest were excluded. The data form, the standard definitions of 87 variables and the registration manual were developed by literature review and the consensus of the study investigators. The registry data were collected by medical chart or electronic medical record reviews. The collected data at each hospital were verified for completeness by the site principal investigator and were recorded in a web-based data registration system [15] by the site clinical research coordinator. A data manager and three clinical research associates monitored and regularly reviewed data quality. The site principal investigators or site clinical research coordinators were contacted through the query function in the system or directly by telephone to clarify the data.

The institutional review board of each institution approved the study protocol before data collection (Additional file 1). Informed consent was waived because of the retrospective nature of the study.

### Study patients and variables

This study included patients who received TH using surface or endovascular cooling devices equipped with an automatic temperature feedback system. Cooling methods without an automatic temperature feedback system such as a simple cooling mattress, extracorporeal membrane oxygenation, cold saline infusion or ice-pack application were included in the study only when they were combined with an automatic temperature feedback device. In contrast, patients who received both types of cooling methods (that is, surface and endovascular) were excluded from this study. Patients who received TH by surface cooling methods, such as hydrogel pads, body wraps and other mattresses, were assigned to the surface cooling group, whereas those who received TH via endovascular cooling catheter were assigned to the endovascular cooling group.

Data included covariates, such as basic demographics, resuscitation variables and post-resuscitation variables. Given that facilities with high volumes of TH cases might demonstrate significantly better outcomes for OHCA patients than those with low volumes [16], the TH volumes of hospitals in the study period were evaluated. Neurological outcomes were assessed just before hospital discharge and were categorized according to the Glasgow–Pittsburgh Cerebral Performance Categories [17]. Variables of cooling efficacy included the induction time (estimated time from the start of cooling to the target temperature) and the rewarming time (estimated time from the start of rewarming to achieving the normo-temperature). Adverse events related to the cooling phase included overcooling (<32°C), bradycardia (<40 beats/minute), tachyarrhythmia, hypokalemia (<3.0 mEq/l), hyperglycemia (>180 mg/dl), bleeding and hypotension. Rebound hyperthermia (>38°C), arrhythmia, hyperkalemia (>5.0 mEq/l), hypoglycemia (<80 mg/dl), bleeding and hypotension were recorded in the rewarming-related adverse events. Tachyarrhythmia during cooling was defined as newly developed tachyarrhythmia during cooling, except for sinus tachycardia. Bleeding was defined as bleeding at any site associated with cooling and rewarming. Hypotension was defined as a systolic blood pressure <90 mmHg or mean arterial pressure <60 mmHg for at least 30 minutes or as the need for supportive measures to maintain a systolic blood pressure >90 mmHg or mean arterial pressure >60 mmHg during cooling and rewarming. Sepsis was defined as a clinical syndrome characterized by the presence of both infection and a systemic inflammatory response syndrome. Pneumonia was defined by the following four findings: new or progressive consolidation on the chest radiograph, fever, leukocytosis and the presence of purulent tracheobronchial secretions.

### Outcome measures

The primary outcome was a comparison of the proportion of patients who had a poor neurological outcome (Cerebral Performance Categories 3 to 5) at discharge. The secondary outcomes were comparisons of the proportion of hospital mortality, induction time and rewarming time as well as the incidence of adverse events during cooling, rewarming and critical care. To adjust for differences in the baseline characteristics of each group, we performed one-to-one matching using the propensity score. Finally, the outcomes were reevaluated in the propensity score matched cohort.

### Statistical analysis

Categorical variables are presented as the counts and percentage and were compared using the chi-square test or Fisher’s exact test, where appropriate. The normality of the continuous variables was verified by the Shapiro–Wilk test. Thereafter, variables were expressed as the mean ± standard deviation and were compared using Student’s *t* test, after confirming homogeneity of variance with Levene’s test. We performed rigorous adjustments for differences in the baseline characteristics of patients using the propensity score. We calculated the propensity score using multivariate logistic regression to model the dichotomous outcome of the surface or the endovascular group for 803 patients in the sample. The logistic model demonstrated a sufficient ability to differentiate between the two groups (*c* statistic = 0.8). We performed one-to-one matching with the propensity score using the Greedy-matching macro [18]. After propensity score matching, the success of the propensity score modeling was assessed by the standardized difference, and the balance of the two groups was evaluated using Student’s *t* test for continuous variables and the chi-square test or Fisher’s exact test for categorical variables. After estimating the propensity scores, we performed a logistic regression analysis to determine the prognosis factor (mortality, poor outcome). Statistical analyses were conducted in SAS (version 9.2; SAS Institute, Inc., Cary, NC, USA). A probability value of *P* <0.05 was considered significant.

## Results

### Characteristics of the study population

**Figure 1:**
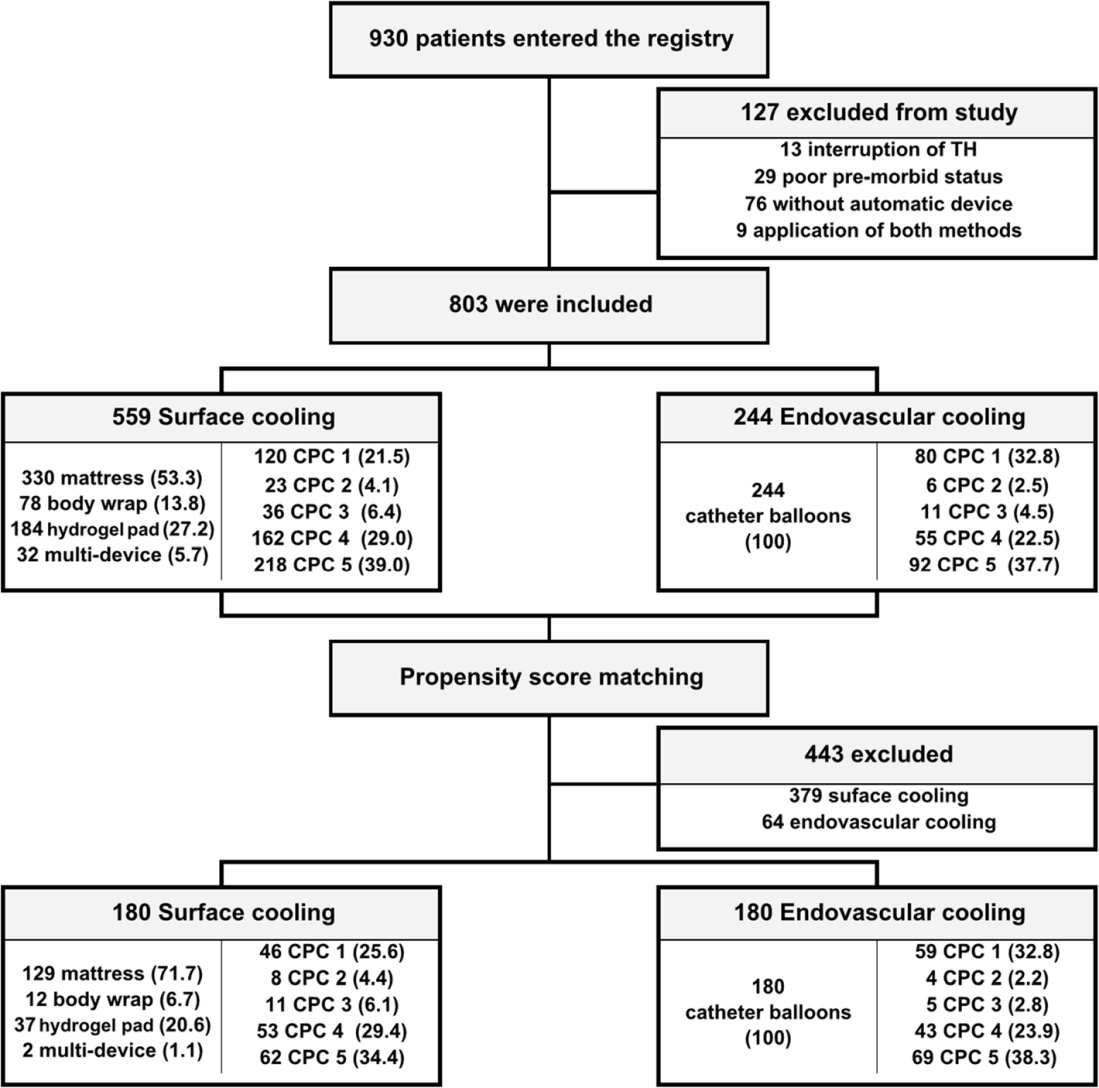
**Flow chart for the inclusion of study patients.** Parentheses indicate a percentage. CPC, Cerebral Performance Category; TH, therapeutic hypothermia.

**Figure 2:**
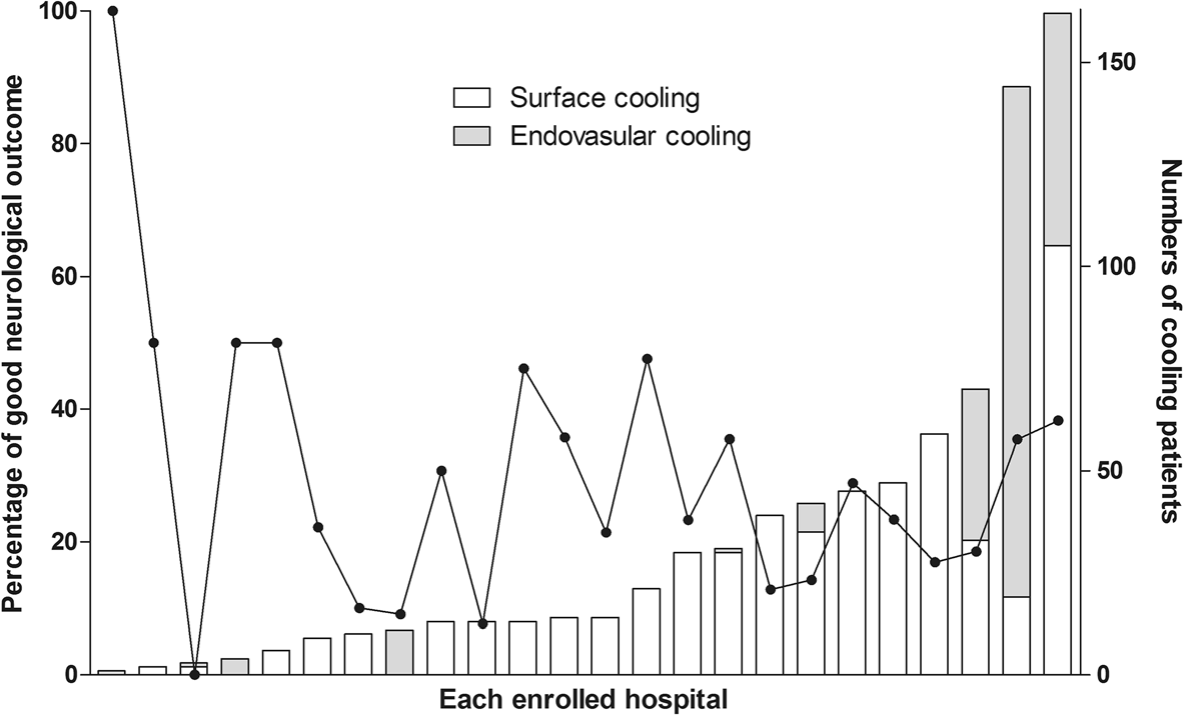
**Choice of cooling methods and neurologic outcome for each hospital.** Stacked bars indicate the number of cooling patients in each enrolled hospital; endovascular cooling (shaded bars) and surface cooling (unshaded bars). The points and connecting line indicate the percentages of good neurological outcome in each enrolled hospital.

Of the 930 OHCA patients entered in the registry between January 2007 and December 2012, 803 were included in the analysis. Of these patients, 559 exclusively underwent surface cooling and the remaining 244 patients underwent endovascular cooling (Figure 1). In the surface cooling group, a cooling mattress (Blanketrol®; CSZ, Louisville, CO, USA or Medi-Therm®; Gaymar, Orchard Park, NY, USA) was the most commonly used device (*n* = 298, 53.3%), followed by a hydrogel pad (Arctic Sun®; Medivance Corp, Louisville, CO, USA) (*n* = 152, 27.2%) and a cooling body wrap (Blanketrol® or Medi-Therm®) (*n* = 77, 13.8%). Multiple surface cooling devices were used in 32 patients. All endovascular cooling groups used heat-exchange catheters with inflatable balloons at the tip (Thermogard XP®; Zoll, Chelmsford, MA, USA). Figure 2 presents the choice of methods and neurologic outcomes for each hospital. The baseline characteristics of the study patients, according to the cooling method, are presented in Table 1.

**Table 1:** **Baseline characteristics of the patients in the overall cohort and in the propensity-matched cohort**

	**Baseline characteristics**					**After propensity matching**				
	**Total**	**Surface group**	**Endovascular group**	***P*** **value**	**STD** ^**a**^	**Total**	**Surface group**	**Endovascular group**	***P*** **value**	**STD** ^**b**^
	**(** ***N*** **= 803)**	**(** ***n*** **= 559)**	**(** ***n*** **= 244)**			**(** ***N*** **= 360)**	**(** ***n*** **= 180)**	**(** ***n*** **= 180)**		
Demographic characteristics										
Male	561 (69.9)	391 (70.0)	170 (69.7)	0.94	−0.65	244 (67.8)	119 (66.1)	125 (69.4)	0.50	7.06
Age (years)	56.9 ± 16.1	57.2 ± 16.1	56.0 ± 16.0	0.32	6.10	55.7 ± 16.9	56.0 ± 17.2	55.5 ± 16.6	0.78	2.40
Underlying disease										
Ischemic heart disease	102 (12.7)	71 (12.7)	31 (12.7)	1.00	0.00	48 (13.3)	23 (12.8)	25 (13.9)	0.76	3.23
Cerebrovascular disease	33 (4.1)	24 (4.3)	9 (3.7)	0.69	−3.06	18 (5.0)	11 (6.1)	7 (3.9)	0.33	−10.11
Hypertension	270 (33.6)	192 (34.4)	78 (32.0)	0.51	−5.10	119 (33.1)	61 (33.9)	58 (32.2)	0.74	−3.61
Diabetes mellitus	180 (22.4)	118 (21.1)	62 (25.4)	0.18	10.19	86 (23.9)	44 (24.4)	42 (23.3)	0.81	−2.58
Lung disease	51 (6.4)	37 (6.6)	14 (5.7)	0.64	−3.75	21 (5.8)	11 (6.1)	10 (5.6)	0.82	−2.13
Renal disease	52 (6.5)	33 (5.9)	19 (7.8)	0.32	7.53	27 (7.5)	14 (7.8)	13 (7.2)	0.84	−2.28
CPR variables										
Witnessed	541 (67.5)	364 (65.2)	177 (72.5)	0.04	15.81	244 (67.8)	122 (67.8)	122 (67.8)	1.00	0.00
Bystander CPR	245 (31.9)	151 (27.9)	94 (41.2)	<0.01	28.25	88 (26.8)	42 (25.6)	46 (27.9)	0.64	5.20
Shockable rhythm	199 (24.9)	125 (22.5)	74 (30.6)	0.02	18.42	85 (23.9)	38 (21.4)	47 (26.4)	0.26	11.74
Anoxic time^c^ (minutes)	32.6 ± 18.4	32.8 ± 18.9	32.1 ± 17.4	0.65	3.10	32.8 ± 19.1	32.7 ± 20.1	32.9 ± 18.1	0.93	−0.84
Cardiac cause	498 (62.0)	335 (59.9)	163 (66.8)	0.07	14.36	231 (64.2)	114 (63.3)	117 (65.0)	0.74	3.55
Variables after ROSC										
STEMI	73 (9.1)	48 (5.6)	25 (10.3)	0.45	5.81	27 (7.5)	14 (7.8)	13 (7.2)	0.84	−2.28
Spontaneous breathing	325 (41.4)	196 (35.8)	129 (54.2)	<0.01	37.63	182 (51.7)	94 (53.4)	88 (50.0)	0.52	−6.81
Motor response	135 (17.0)	83 (14.9)	52 (21.9)	0.02	18.14	71 (19.9)	37 (20.7)	34 (19.1)	0.71	−4.01
Pupil light reflex	343 (43.4)	238 (42.9)	105 (44.7)	0.64	3.63	174 (49.7)	88 (50.0)	86 (49.4)	0.91	−1.20
Hospital variable										
TH volume	90.2 ± 61.5	70.4 ± 55.2	135.6 ± 50.0	<0.01	−99.46	129.4 ± 55.9	130.1 ± 58.5	128.6 ± 53.3	0.80	2.16

Continuous variables are presented as the mean ± standard deviation. Parentheses indicate a percentage. CPR, cardiopulmonary resuscitation; ROSC, return of spontaneous circulation; STEMI, ST segment elevation myocardial infarction; TH, therapeutic hypothermia. ^a^Standardized difference between the surface group and endovascular group before propensity score matching.

^b^Standardized difference between the surface group and endovascular group after propensity score matching. ^c^Anoxic time is the time from cardiac arrest to return of spontaneous circulation.

No differences in age, sex or underlying disease were observed between the two groups. However, patients receiving endovascular cooling were significantly more likely to be witnessed during cardiac arrest, to display an increased bystander cardiopulmonary resuscitation rate and to exhibit a greater number of shockable rhythms than surface cooling patients (72.5% vs. 65.2%, *P* = 0.04, 41.2% vs. 27.9%, *P* <0.01 and 30.6% vs. 22.5%, *P* = 0.02 respectively). More patients in the endovascular cooling group experienced spontaneous breathing and motor response immediately after return of spontaneous circulation compared with the surface cooling group (54.2% vs. 35.8%, *P* <0.01 and 21.9% vs. 14.9%, *P* = 0.12 respectively). The number of patients treated by TH during the study period was significantly larger in the hospitals of the endovascular cooling group than in the hospitals of the surface cooling group (135.6 ± 50.0 vs. 70.4 ± 55.2, *P* <0.01).

### Outcomes for unmatched patients

Table 2 presents the cooling efficacy, adverse events and neurological outcomes according to the cooling methods in the overall cohort. Induction time and rewarming time were not associated with the cooling method; however, a rewarming time that was slower than the median time (≥720 minutes) was significantly associated with endovascular cooling (*P* = 0.04).

**Table 2:** **Cooling efficacy, complication and neurological outcomes between the surface cooling and endovascular cooling groups**

	**Total (** ***N*** **= 803)**	**Surface group**	**Endovascular group**	***P*** **value**
		**(** ***n*** **= 559)**	**(** ***n*** **= 244)**	
Time variables				
Induction time (minutes)	231.1 ± 9.1	240.1 ± 11.8	211.1 ± 12.9	0.10
≥165 minutes (cutoff value: median)	395 (51.1)	269 (50.3)	126 (52.9)	0.50
Rewarming time (minutes)	745.9 ± 17.8	744.3 ± 23.1	749.5 ± 25.7	0.88
≥720 minutes (cutoff value: median)	353 (50.4)	232 (47.8)	121 (56.3)	0.04
Adverse events in the cooling phase				
Overcooling	151 (18.8)	131 (23.5)	20 (8.2)	<0.01
Bradycardia	106 (13.3)	84 (15.1)	22 (9.0)	0.02
Tachycardia	99 (12.4)	81 (14.5)	18 (7.5)	0.01
Hypokalemia	234 (29.3)	170 (30.6)	64 (26.3)	0.22
Hyperglycemia	369 (46.1)	295 (53.0)	74 (30.3)	<0.01
Bleeding	26 (3.3)	20 (3.6)	6 (2.5)	0.42
Hypotension	280 (35.0)	209 (37.6)	71 (29.1)	0.02
Adverse events in the rewarming phase				
Hyperthermia	80 (10.7)	67 (13.0)	13 (5.6)	<0.01
Arrhythmia	42 (5.7)	37 (7.2)	5 (2.2)	0.01
Hyperkalemia	56 (7.5)	39 (7.6)	17 (7.4)	0.91
Hypoglycemia	73 (9.8)	55 (10.7)	18 (7.8)	0.22
Bleeding	12 (1.6)	7 (1.4)	5 (2.2)	0.53
Hypotension	180 (24.2)	133 (25.9)	47 (20.4)	0.10
Adverse events in critical care				
Sepsis	59 (7.5)	40 (7.3)	19 (7.8)	0.80
Pneumonia	283 (35.6)	191 (34.6)	92 (38.0)	0.36
Neurological outcome				
Hospital mortality	310 (38.6)	218 (39.0)	92 (37.7)	0.73
Poor neurological outcome	574 (71.5)	416 (74.4)	158 (64.7)	0.01

Continuous variables are presented as the mean ± standard error. Parentheses indicate a percentage.

More adverse events, such as overcooling, arrhythmia, hyperglycemia or hypotension in the cooling phase and hyperthermia and arrhythmia in the rewarming phase, occurred in the surface cooling group, which was significantly associated with a poor neurological outcome at hospital discharge (odds ratio (OR): 1.58, 95% confidence interval (CI): 1.15 to 2.19, *P* <0.01) (Table 3).

**Table 3:** **Independent predictors for poor neurological outcome and hospital discharge in the unmatched cohort**

	**Poor neurological outcome**						**Hospital mortality**					
	**OR**	**95% CI**	***P*** **value**	**Adjusted OR**	**95% CI**	***P*** **value**	**OR**	**95% CI**	***P*** **value**	**Adjusted OR**	**95% CI**	***P*** **value**
Method												
Surface cooling	1.58	1.15 to 2.19	0.01	1.46	0.81 to 2.63	0.21	1.06	0.78 to 1.44	0.73	0.78	0.50 to 1.21	0.26
Demographic characteristics												
Male	0.50	0.35 to 0.72	<0.01	0.63	0.37 to 1.06	0.08	0.56	0.41 to 0.76	<0.01	0.67	0.47 to 0.96	0.03
Age	1.04	1.03 to 1.05	<0.01	1.04	1.02 to 1.06	<0.01	1.02	1.01 to 1.03	<0.01	1.02	1.00 to 1.03	0.01
≥57 years old	3.04	2.20 to 4.20	<0.01				1.92	1.44 to 2.57	<0.01			
Underlying disease												
Ischemic heart disease	0.77	0.50 to 1.20	0.25				0.85	0.55 to 1.31	0.46			
Cerebrovascular disease	2.30	0.88 to 6.03	0.09				1.96	0.98 to 3.96	0.06			
Hypertension	1.63	1.16 to 2.28	0.01	1.29	0.72 to 2.31	0.40	1.29	0.95 to 1.73	0.10			
Diabetes mellitus	2.59	1.67 to 4.00	<0.01	1.68	0.86 to 3.29	0.13	1.94	1.39 to 2.72	<0.01	1.54	1.02 to 2.33	0.04
Lung disease	5.02	1.79 to 14.09	<0.01	3.09	0.82 to 11.65	0.10	1.22	0.69 to 2.17	0.49			
Renal disease	5.13	1.83 to 14.41	<0.01	1.48	0.43 to 5.13	0.54	2.11	1.20 to 3.72	0.01	1.09	0.56 to 2.15	0.80
CPR variables												
Witnessed	0.36	0.25 to 0.53	<0.01	0.58	0.34 to 1.01	0.05	0.43	0.31 to 0.58	<0.01	0.60	0.42 to 0.85	0.01
Bystander CPR	1.96	1.41 to 2.72	<0.01	1.22	0.72 to 2.04	0.46	0.78	0.57 to 1.07	0.12			
Shockable rhythm	0.51	0.37 to 0.71	<0.01	0.51	0.30 to 0.87	0.01	0.33	0.22 to 0.48	<0.01	0.62	0.39 to 0.99	0.05
Anoxic time^a^	1.03	1.02 to 1.04	<0.01	1.05	1.03 to 1.06	<0.01	1.02	1.01 to 1.03	<0.01	1.02	1.01 to 1.03	<0.01
≥30 minutes	2.64	1.92 to 3.63	<0.01				1.83	1.37 to 2.45	<0.01			
Cardiac cause	0.14	0.09 to 0.22	<0.01	0.19	0.11 to 0.35	<0.01	0.37	0.28 to 0.50	<0.01	0.55	0.38 to 0.79	<0.01
Variables after ROSC												
STEMI	0.35	0.21 to 0.57	<0.01	0.78	0.38 to 1.60	0.49	0.66	0.39 to 1.12	0.12			
Spontaneous breathing	0.19	0.14 to 0.27	<0.01	0.41	0.25 to 0.67	<0.01	0.27	0.20 to 0.38	<0.01	0.46	0.32 to 0.68	<0.01
Motor response	0.10	0.07 to 0.15	<0.01	0.23	0.13 to 0.41	<0.01	0.16	0.09 to 0.28	<0.01	0.45	0.24 to 0.85	0.01
Pupil light reflex	0.11	0.07 to 0.15	<0.01	0.26	0.16 to 0.42	<0.01	0.21	0.15 to 0.30	<0.01	0.43	0.30 to 0.64	<0.01
Hospital variable												
TH volume	1.00	0.99 to 1.00	<0.01	1.00	1.00 to 1.01	0.21	1.00	0.99 to 1.00	<0.01	1.00	1.00 to 1.00	0.34
≥60	0.63	0.46 to 0.86	<0.01				0.77	0.58 to 1.03	0.08			

CI, confidence interval; CPR, cardiopulmonary resuscitation; OR, odds ratio; ROSC, return of spontaneous circulation; STEMI, ST segment elevation myocardial infarction; TH, therapeutic hypothermia. ^a^Anoxic time is the time from cardiac arrest to return of spontaneous circulation.

The results of the multivariable analysis are illustrated in Table 3. Using logistic regression and adjusting for potential outcome predictors (including the cooling method, sex, age, underlying disease, cardiopulmonary resuscitation variables, variables after return of spontaneous circulation and hospital TH volume), we observed that the surface cooling method was not an independent predictor of poor neurological outcome (OR: 1.46, 95% CI: 0.81 to 2.63, *P* = 0.21).

### Characteristics of patients matched for propensity scores

After propensity score matching was performed for the entire population, 180 matched pairs of patients were available (Table 1, after propensity matching). In the matched cohorts, no significant differences between both groups for any of the covariates were noted.

### Outcomes for matched patients

**Figure 3:**
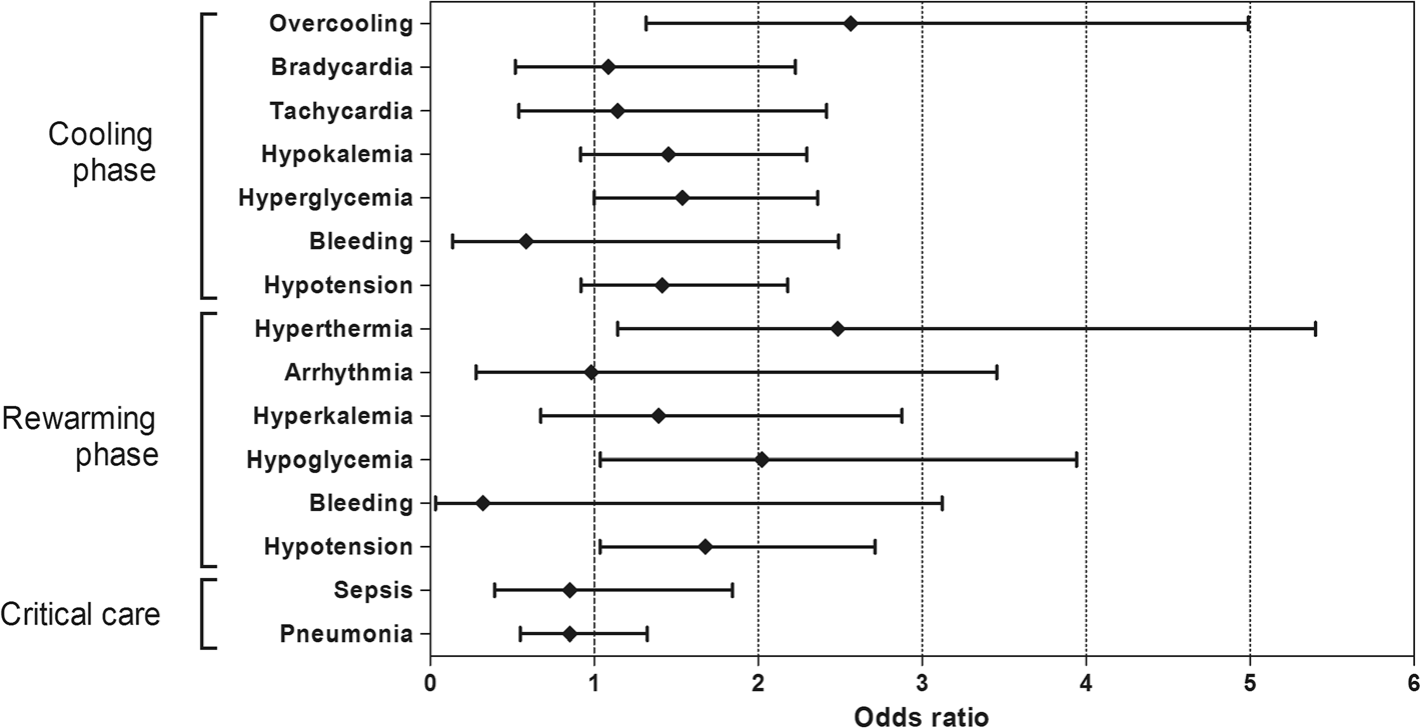
**Odds ratio for each adverse event for surface cooling.** Each plot represents the odds ratio and 95% confidence interval.

In the outcome analysis (Table 4), we observed no significant difference in the neurological outcomes between the surface cooling and endovascular cooling groups. The use of surface cooling methods was not a predictor for poor neurological outcome or hospital mortality (OR: 1.26, 95% CI: 0.81 to 1.96, *P* = 0.31 and OR: 0.85, 95% CI: 0.55 to 1.30, *P* = 0.44 respectively). With regard to cooling efficacy, both groups spent a similar amount of time attaining the target temperature, but the surface cooling group rewarmed at a more rapid rate (625.0 ± 34.9 minutes vs.741.3 ± 32.3 minutes, *P* <0.01). The rates of some adverse events were significantly increased in the surface cooling group compared with the endovascular cooling group. The ORs of surface cooling for overcooling, rebound hyperthermia and rewarming-related hypoglycemia and hypotension were 2.56 (95% CI: 1.32 to 4.99, *P* = 0.01), 2.49 (95% CI: 1.15 to 5.40, *P* = 0.02), 2.02 (95% CI 1.04 to 3.94, *P* = 0.04) and 1.68 (95% CI: 1.04 to 2.71, *P* = 0.04), respectively (Figure 3).

**Table 4:** **Logistic regression analysis of the matched cohort for outcomes, efficacies and complications**

	**Surface cooling group**	**Endovascular cooling group**	**Odds ratio**	**95% confidence interval**	***P*** **value**
	**(** ***n*** **= 180)**	**(** ***n*** **= 180)**			
Time variables					
Induction time (minutes)	235.3 ± 18.0	209.4 ± 15.4	1.13	0.79 to 1.62	0.51
≥173.5 minutes (cutoff value: median)	88 (50.0)	89 (50.0)	1.00	0.66 to 1.52	1.00
Rewarming time (minutes)	625.0 ± 34.9	741.3 ± 32.3	0.50	0.34 to 0.73	<0.01
≥660 minutes (cutoff value: median)	68 (40.7)	103 (65.2)	0.37	0.23 to 0.58	<0.01
Adverse events in the cooling phase					
Overcooling	32 (17.8)	14 (7.8)	2.56	1.32 to 4.99	0.01
Bradycardia	16 (9.0)	15 (8.3)	1.09	0.52 to 2.27	0.83
Tachycardia	16 (8.9)	14 (7.9)	1.14	0.54 to 2.42	0.73
Hypokalemia	59 (32.8)	45 (25.1)	1.45	0.92 to 2.30	0.11
Hyperglycemia	104 (57.8)	58 (32.2)	1.54	1.00 to 2.36	0.05
Bleeding	3 (1.7)	5 (2.9)	0.59	0.14 to 2.49	0.45
Hypotension	72 (40.2)	58 (32.2)	1.42	0.92 to 2.18	0.12
Adverse events in the rewarming phase					
Hyperthermia	23 (13.5)	10 (5.9)	2.49	1.15 to 5.40	0.02
Arrhythmia	5 (2.9)	5 (3.0)	0.98	0.28 to 3.46	0.98
Hyperkalemia	19 (11.1)	14 (8.2)	1.39	0.67 to 2.88	0.37
Hypoglycemia	28 (16.4)	15 (8.8)	2.02	1.04 to 3.94	0.04
Bleeding	1 (0.6)	3 (1.8)	0.32	0.03 to 3.12	0.33
Hypotension	56 (32.6)	38 (22.4)	1.68	1.04 to 2.71	0.04
Adverse events in critical care					
Sepsis	13 (7.2)	15 (8.4)	0.85	0.39 to 1.84	0.68
Pneumonia	57 (31.8)	63 (35.4)	0.85	0.55 to 1.32	0.48
Neurological outcome					
Hospital mortality	62 (34.4)	69 (38.3)	0.85	0.55 to 1.30	0.44
Poor neurological outcome	126 (70.0)	117 (65.0)	1.26	0.81 to 1.96	0.31

Continuous variables are presented as the mean ± standard error. Parentheses indicate a percentage.

### Comparisons between hydrogel pads and catheter balloons

Multiple types of surface cooling methods were included in this study, whereas a heat-exchange catheter with saline-circulating balloons was the only endovascular cooling method device. To eliminate the influence of technological diversity, only one type of modern surface cooling device using hydrogel pads was compared with the endovascular cooling device. Similar to the preceding analysis, propensity score matching was performed in patients treated with both devices. Ultimately, 59 matched pairs of patients were available. There were no significant differences in hospital mortality and poor neurological outcome (Table 5).

**Table 5:** **Logistic regression analysis of the matched cohort of hydrogel pads and catheter balloons for outcomes, efficacies and complications**

	**Hydrogel pads**	**Catheter balloons**	**Odds ratio**	**95% confidence interval**	***P*** **value**
	**(** ***n*** **= 59)**	**(** ***n*** **= 59)**			
Time variables					
Induction time (minutes)	188.1 ± 22.6	243.4 ± 35.3	0.73	0.39 to 1.39	0.34
≥160.0 minutes (cutoff value: median)	26 (45.6)	32 (56.1)	0.66	0.31 to 1.37	0.26
Rewarming time (minutes)	923.2 ± 61.1	697.3 ± 67.7	3.23	1.60 to 6.52	<0.01
≥720 minutes (cutoff value: median)	39 (78.0)	23 (43.40)	4.62	1.95 to 10.95	<0.01
Adverse events in the cooling phase					
Overcooling	8 (13.56)	9 (15.25)	0.87	0.31 to 2.44	0.79
Bradycardia	9 (15.25)	7 (11.86)	1.34	0.46 to 3.86	0.59
Tachycardia	7 (11.86)	11 (19.30)	0.56	0.20 to 1.57	0.27
Hypokalemia	16 (27.12)	13 (22.41)	1.29	0.56 to 2.99	0.56
Hyperglycemia	34 (57.63)	27 (45.76)	1.61	0.78 to 3.34	0.20
Bleeding	3 (5.08)	3 (5.45)	0.93	0.18 to 4.81	0.93
Hypotension	25 (42.37)	17 (28.81)	1.82	0.85 to 3.90	0.13
Adverse events in the rewarming phase					
Hyperthermia	7 (12.73)	1 (1.82)	7.88	0.94 to 66.33	0.06
Arrhythmia	4 (7.41)	3 (5.66)	1.33	0.28 to 6.27	0.72
Hyperkalemia	6 (11.11)	6 (10.91)	1.02	0.31 to 3.39	0.97
Hypoglycemia	8 (14.81)	2 (3.64)	4.61	0.93 to 22.81	0.06
Bleeding	2 (3.70)	2 (3.85)	0.96	0.13 to 7.09	0.97
Hypotension	19 (35.19)	12 (21.82)	1.95	0.83 to 4.55	0.13
Adverse events in critical care					
Sepsis	1 (1.69)	8 (13.79)	0.11	0.01 to 0.89	0.04
Pneumonia	17 (28.81)	22 (37.93)	0.66	0.31 to 1.44	0.30
Neurological outcome					
Hospital mortality	26 (44.07)	22 (37.29)	1.33	0.63 to 2.77	0.45
Poor neurological outcome	45 (76.27)	40 (67.80)	1.53	0.68 to 3.44	0.31

Continuous variables are presented as the mean ± standard error. Parentheses indicate a percentage.

Unlike the overall matched cohort, the hydrogel pad group rewarmed at a slower rate (923.2 ± 61.1 minutes vs. 697.3 ± 67.7 minutes, *P* <0.01), and the OR of the hydrogel pad group for sepsis was 0.11 (95% CI: 0.01 to 0.89, *P* = 0.04). Overcooling, rebound hyperthermia and rewarming-related hypoglycemia and hypotension were not associated with the use of hydrogel pads.

## Discussion

We compared neurological outcomes, efficacies and adverse events between surface and endovascular cooling techniques. Our multicenter, retrospective, observational, registry-based study indicated that the rates of poor neurological outcome and hospital mortality were similar in both groups; however, the incidences of certain adverse events were more common in the surface cooling group.

Our findings are consistent with those published in the literature. Gillies and colleagues reported no difference in hospital mortality and neurological outcomes between the two groups; in addition, endovascular cooling provides improved temperature management compared with surface cooling as well as a more favorable complication profile [12]. However, this study only compared the use of endovascular cooling using automatic temperature control feedback with ice bags without the aid of temperature feedback regulation. In a single-center observational study, similar survival-to-hospital discharge and comparable neurological outcomes were observed in the endovascular cooling group using catheter balloons and in the surface cooling group using hydrogel pads [13]. Recently, one randomized controlled trial reported that invasive endovascular cooling has advantages over surface cooling using hydrogel pads with respect to temperature management, but these techniques did not result in a different outcome [14].

There are known differences between surface and endovascular cooling. Some of the literature has reported a significant increase in the rate of the occurrence of shivering in patients treated with surface cooling devices. Surface cooling is known to cause shivering, resulting in possible sympathetic activation [19-22]. If shivering is not controlled, the body temperature will not decrease, despite cooling. In contrast, hypothermia induced by core cooling with cardiopulmonary bypass produces sympathetic inhibition in anesthetized rabbits [23]. In our study, paralyzing agents were generally used but were selected case by case. Therefore, we could not include shivering as a meaningful adverse event. Surface cooling techniques using non-invasive devices are easy to implement and have advantages, such as the lack of the requirement for puncturing an additional main vessel. This feature may reduce complications and infections in resuscitated patients, who represent a highly vulnerable population [6]. Endovascular cooling involves the insertion of a large gauge catheter into the vena cava, which is an invasive and time-consuming procedure [10,14]. In contrast, surface cooling techniques can be labor intensive for the nursing staff and might prevent access to the patient [12]. Despite these findings, the best method for treatment in a clinical setting has not been identified.

Various possible factors might influence the neurological outcome of cardiac arrest patients treated with TH. The first possible factor is time to target temperature. Evidence suggests that the time to TH has an important impact on the outcome [8,24,25]. Given that surface cooling increases the rate of shivering [19-22] and some simple surface devices are less effective at cooling than endovascular cooling [26], the time needed from the start of cooling to achieve the target temperature was significantly increased for surface cooling [14]. The time required to initiate treatment is another factor to be considered when comparing endovascular cooling with surface cooling. Inserting an endovascular device requires the availability of medical personnel, whereas surface cooling can be initiated quickly by the nursing staff [27]. In contrast, although the use of early, prehospital cooling reduced the core temperature by the time of hospital arrival and reduced the time to reach 34°C, it did not improve survival or functional status among patients resuscitated from OHCA in a recent randomized clinical trial of the prehospital induction of mild hypothermia [28]. Second, surface cooling is associated with failure to achieve the target temperature (33°C) and with overcooling [29,30]. A new randomized trial reported no significant difference between a near-normal temperature (36°C) and induced hypothermia (33°C) [31]. One interpretation of these results is that active prevention of hyperthermia is more important than a strict lower temperature or early induction in cardiac arrest patients. This finding potentially explains why the surface cooling method, which is associated to a greater extent with poor temperature management, is not inferior to the core cooling method with respect to neurological outcomes.

Interestingly, a higher rate of some adverse events in the surface cooling group was observed compared with the endovascular cooling group in the matched cohort. However, many wide confidence intervals reflected the small sample size; thus, these results may not show a significant difference between the two groups.

Patients receiving surface cooling might be at increased risk of overcooling and rebound hyperthermia as a result of problems with the equilibration of the peripheral temperature with the core body temperature [29,32]. Hypoglycemia is also a well-known rewarming-related adverse event [33,34], and more rapid rewarming and rebound hyperthermia in the surface cooling group might lead to these rewarming-related adverse events. The application of external heat may cause peripheral vasodilation and venous pooling, leading to relative hypovolemia and hypotension [35]. In a previous report regarding complications of TH, bleeding and sepsis that occurred after invasive procedures, such as those using intravascular devices for cooling, as well as sustained hyperglycemia were associated with increased mortality [36]. However, these adverse events that were known to be associated with worse outcomes were not associated with specific cooling methods in our study.

Several of our findings deserve further mention. First, in the unmatched cohort, patients receiving endovascular cooling exhibited good clinical variables and were also more likely to be treated in a high TH volume hospital. This finding was significantly associated with good neurological outcomes. We therefore performed one-to-one matching using the propensity score. After propensity score matching, the two groups were balanced. Second, we compared all of the surface cooling devices, including hydrogel pads and many of the simple water circulating blankets/wraps, with endovascular cooling devices. This study is the first to compare overall surface cooling devices with endovascular cooling devices. Endovascular cooling is a unique technique that uses an automatic temperature control feedback system. However, surface cooling devices are diverse. Hydrogel pads differ from other cooling blankets by producing higher cold fluid flow rates, utilizing conductive, adherent gel pads and implementing a precise temperature feedback-control mechanism. These factors might allow for a more rapid induction of cooling and improved control of temperature during hypothermia maintenance, as well as rewarming, than other cooling blankets. However, in a recent study comparing hydrogel pads and a conventional standard cooling blanket, no differences in the incidence of complications during TH and neurological outcomes were reported between the two groups [37]. In our study, more than two-thirds of patients who were cooled by these other devices were included in the surface cooling group. Nevertheless, no difference was observed in the neurological outcomes between the two groups, while some adverse events were associated with the surface cooling group. However, when we confine the surface cooling device to hydrogel pads, these adverse events were no longer observed. The hydrogel pad group rewarmed more slowly but within the range of the targeted rewarming rate (0.2 to 0.3°C/hour). The OR of the hydrogel pad group for sepsis had a wide CI (0.01 to 0.89), which implies low statistical power due to limited numbers of matched patients. Our results may indicate that some adverse events in the surface cooling group were not the result of differences between both methods, but were caused by an inability to precisely control the body temperature. Furthermore, these differences did not have an effect on the neurological outcome.

### Limitations

The results of this study should be interpreted in the context of several limitations. A major limitation of this study was the possibility of selection bias and reporting bias. Although we tried to mimic randomization, there was an inevitable risk of bias because our study was a retrospective, registry-based multicenter study. Additionally, our retrospective registry did not include potential confounding factors such as cardiac intervention, hemodynamic status, time of endovascular catheter insertion and various complications regarding vascular access. Because this limitation can lead to a skewed perception of the adverse event rates between the two groups, our results must be interpreted with caution. Although we adjusted for hospital TH volume as a potential confounder, the majority of hospitals used either surface or endovascular cooling, and we did not adjust for other hospital factors. Variable TH protocols or differences in care among hospitals may therefore affect the incidence of adverse events, time variables and outcome. Although this was a multicenter registry study, the sample size was relatively small after we performed one-to-one matching, and some wide ORs reflected low statistical power. Finally, in our registry, only one type of endovascular device was used whereas the surface cooling group included various devices.

## Conclusions

In the matched cohort, no significant differences in the rates of poor neurological outcomes and hospital mortality were observed between the surface cooling and endovascular cooling groups. Although overcooling, rebound hyperthermia, rewarming-related hypoglycemia and rewarming-related hypotension were significantly increased in the surface cooling group compared with the endovascular cooling group, these complications were not associated with the surface cooling method using hydrogel pads.

## Key messages

- No significant difference in neurological outcome and hospital mortality was observed between the surface and endovascular cooling methods.
- Overcooling, rebound hyperthermia, rewarming-related hypoglycemia and rewarming-related hypotension were significantly increased in the surface cooling group compared with the endovascular cooling group.
- These adverse events of surface cooling were not observed in the modern surface cooling method using hydrogel pads.

## Additional file

Additional file 1:
**Is a table presenting a list of the contributing sites and their ethical approval bodies.**

## Abbreviations

CI: confidence interval
OHCA: out-of-hospital cardiac arrest
OR: odds ratio
TH: therapeutic hypothermia
