# Supplementary material for: An observational study of surface versus endovascular cooling techniques in cardiac arrest patients: a propensity-matched analysis
Source: Crit Care. 2015 Mar 16;19(1):85. doi: 10.1186/s13054-015-0819-7 (PMC4367874; doi:10.1186/s13054-015-0819-7)
Supplement: Additional file 1: — Is a table presenting a list of the contributing sites and their ethical approval bodies. [file 13054_2015_819_MOESM1_ESM.docx]

| **City** | **Hospital** | **Principle Investigator** |
| --- | --- | --- |
| Anyang | Hallym University Sacred Heart Hospital | Yoo Dong Son |
| Bucheon | Soonchunhyang University Hospital | Hoon Lim |
| Cheongju | Chungbuk National University Hospital | Jin Hong Min |
| Daegu | Kyungpook National University Hospital | Jung Bae Park |
|  | Daegu Catholic University Medical Center | Tae Chang Jang |
| Daejeon | Chungnam National University Hospital | Yeon Ho You |
| Goyang | Inje University Ilsan Paik Hospital | Kyung Hwan Kim |
| Gwangju | Chonnam National University Hospital | Byung Kook Lee |
| Seoul | Asan Medical Center, University of Ulsan College of Medicine | Won Young Kim |
|  | Chung-Ang University Hospital | Dong Hoon Lee |
|  | Ewha Womans University Mokdong Hospital | Chul Han |
|  | Hallym University Kangdong Sacred Heart Hospital | Gyu Chong Cho |
|  | Hallym University Kangnam Sacred Heart Hospital | Gu Hyun Kang |
|  | Hanil General Hospital, Korea Electric Power Medical Corporation | In Soo Cho |
|  | Kyung Hee University Medical Center | Jong Seok Lee |
|  | Seoul National University Borame Medical Center | Jonghwan Shin |
|  | Seoul National University Hospital | Gil Joon Suh |
|  | Seoul St. Mary’s Hospital, The Catholic University of Korea College of Medicine | Kyu Nam Park |
|  | Severance Hosptial, Yonsei University College of Medicine | Yoo Seok Park |
|  | Yeouido St. Mary’s Hospital, The Catholic University of Korea College of Medicine | Seung Pill Choi |
| Suwon | Ajou University Hospital | Gi Woon Kim |
| Uijeongbu | Uijeongbu St. Mary’s Hospital, The Catholic University of Korea College of Medicine | Joo Suk Oh |
| Ulsan | Ulsan University Hospital | Wook Jin Choi |
| Wonju | Wonju Severance Christian Hospital, Yonsei University Wonju College of Medicine | Kyoung Chul Cha |
